# Enhancing petunia tissue culture efficiency with machine learning: A pathway to improved callogenesis

**DOI:** 10.1371/journal.pone.0293754

**Published:** 2023-11-03

**Authors:** Hamed Rezaei, Asghar Mirzaie-asl, Mohammad Reza Abdollahi, Masoud Tohidfar

**Affiliations:** 1 Department of Plant Biotechnology, Faculty of Agriculture, Bu-Ali Sina University, Hamedan, Iran; 2 Department of Agronomy and Plant Breeding, Faculty of Agriculture, Bu-Ali Sina University, Hamedan, Iran; 3 Department of Plant Biotechnology, Faculty of Life Science and Biotechnology, Shahid Beheshti University, Tehran, Iran; Adamas University, INDIA

## Abstract

The important feature of petunia in tissue culture is its unpredictable and genotype-dependent callogenesis, posing challenges for efficient regeneration and biotechnology applications. To address this issue, machine learning (ML) can be considered a powerful tool to analyze callogenesis data, extract key parameters, and predict optimal conditions for petunia callogenesis, facilitating more controlled and productive tissue culture processes. The study aimed to develop a predictive model for callogenesis in petunia using ML algorithms and to optimize the concentrations of phytohormones to enhance callus formation rate (CFR) and callus fresh weight (CFW). The inputs for the model were BAP, KIN, IBA, and NAA, while the outputs were CFR and CFW. Three ML algorithms, namely MLP, RBF, and GRNN, were compared, and the results revealed that GRNN (R^2^≥83) outperformed MLP and RBF in terms of accuracy. Furthermore, a sensitivity analysis was conducted to determine the relative importance of the four phytohormones. IBA exhibited the highest importance, followed by NAA, BAP, and KIN. Leveraging the superior performance of the GRNN model, a genetic algorithm (GA) was integrated to optimize the concentration of phytohormones for maximizing CFR and CFW. The genetic algorithm identified an optimized combination of phytohormones consisting of 1.31 mg/L BAP, 1.02 mg/L KIN, 1.44 mg/L NAA, and 1.70 mg/L IBA, resulting in 95.83% CFR. To validate the reliability of the predicted results, optimized combinations of phytohormones were tested in a laboratory experiment. The results of the validation experiment indicated no significant difference between the experimental and optimized results obtained through the GA. This study presents a novel approach combining ML, sensitivity analysis, and GA for modeling and predicting callogenesis in petunia. The findings offer valuable insights into the optimization of phytohormone concentrations, facilitating improved callus formation and potential applications in plant tissue culture and genetic engineering.

## Introduction

Plant tissue culture approaches have revolutionized the field of plant propagation and plant improvement due to their remarkable potential for rapid multiplication of plant species, germplasm preservation, and the production of disease-free plant material [1–4]. Petunia (*Petunia hybrida*) is an ornamental flowering plant widely cultivated for its vibrant and diverse flower colors, making it a popular valuable plant in the horticultural industry [5]. Traditional methods of petunia propagation, such as seed germination and vegetative propagation through cuttings, often face limitations in terms of time, efficiency, and genetic stability [6–8]. Therefore, the utilization of plant tissue culture techniques offers valuable alternatives for overcoming these challenges and achieving more efficient and reliable propagation and plant improvement strategies in petunia [9].

One of the key advantages of plant tissue culture approaches in petunia propagation is the ability to achieve rapid and mass multiplication of elite cultivars [6–8]. Through techniques such as shoot proliferation, somatic embryogenesis, and organogenesis, a large number of plantlets can be generated from a single explant, irrespective of the availability of seeds or the season [10–12]. This rapid multiplication not only facilitates commercial production but also aids in the preservation of valuable germplasm and rare genotypes [13, 14]. Furthermore, tissue culture methods allow for the production of disease-free plant material by eliminating pathogens during the sterilization process and maintaining strict aseptic conditions throughout the culture period [15]. The resultant plants are free from diseases such as viruses, bacteria, and fungi, ensuring their healthy establishment in the field or greenhouse [16]. These disease-free plants also serve as a valuable resource for genetic improvement programs, providing a clean starting point for the introduction of desirable traits and the development of novel cultivars in petunia [9]. Overall, plant tissue culture approaches offer significant advantages in terms of rapid multiplication and disease-free propagation, making them indispensable tools in the propagation and plant improvement of petunia [6–8].

Callus formation is a crucial multi-variable process that plays a pivotal role in plant tissue culture systems [17]. Callus, an undifferentiated mass of cells derived from explants, serves as an intermediate stage for subsequent organogenesis or embryogenesis, and is widely utilized for various applications including plant regeneration, genetic transformation, and secondary metabolite production [18]. The formation and development of callus are influenced by a multitude of factors, including explant type, culture medium composition, hormonal balance, physical conditions, and genetic factors [19]. Understanding the intricate interplay among these variables is essential for optimizing callus induction and subsequent plant regeneration protocols. However, due to the complex nature of callus formation, it remains a challenging process to predict and control [20]. This necessitates a comprehensive investigation into the multiple factors governing callus initiation and growth, enabling a better understanding of the underlying mechanisms and facilitating the development of efficient tissue culture systems for a wide range of plant species [21].

Machine learning techniques have emerged as powerful tools for advancing our understanding of complex biological processes, including callus formation in plant tissue culture systems [22–28]. Traditional approaches have relied on empirical observations and trial-and-error methods, which are time-consuming and often yield suboptimal results [29, 30]. In recent years, machine learning algorithms have demonstrated great potential in deciphering the underlying mechanisms of callus formation and facilitating the optimization of tissue culture protocols [31–33]. By leveraging large datasets and complex mathematical models, machine learning algorithms can identify patterns, extract relevant features, and predict outcomes with a higher degree of accuracy [34–37]. These techniques enable researchers to systematically analyze and integrate diverse variables, such as explant type, culture medium composition, hormonal balance, physical conditions, and genetic factors, in order to gain a deeper understanding of their individual and collective contributions to callus induction and growth [20]. Moreover, machine learning algorithms can uncover non-linear relationships and hidden interactions among variables, providing valuable insights into the complex dynamics of callus formation [33]. This knowledge can then be utilized to design targeted experiments, optimize culture conditions, and develop predictive models that enhance the efficiency and reliability of the callus formation process [38]. Overall, the integration of machine learning techniques holds immense promise for advancing our understanding of callus formation mechanisms and facilitating the development of more efficient and precise tissue culture protocols in plant biotechnology [19, 39].

The current study was aimed to harness the power of machine learning techniques to improve our understanding of the complex process of callus formation in *Petunia hybrida* tissue culture systems. Therefore, the specific objectives of this study include: (1) collecting a comprehensive dataset comprising various concentrations of different phytohormones influencing callogenesis; (2) training and validating machine learning algorithms on the dataset to develop predictive models for callus induction and growth in Petunia; (3) identifying key variables and their interactions that significantly influence callus formation; (4) gaining insights into the non-linear relationships and complex dynamics underlying callus formation; and (5) utilizing the developed predictive models to optimize tissue culture protocols and improve the efficiency and reliability of callus formation in Petunia. By achieving these objectives, this study aims to contribute to the field of plant tissue culture and provide valuable tools and knowledge for the advancement of Petunia biotechnology applications.

## Materials and methods

### Plant material, culture condition and treatments

In this study, the plant material used was Petunia (*Petunia hybrida*) cultivar ‘Red Fire chief’. The *in vitro* seed sterilization and germination for obtaining *in vitro*-grown seedlings were performed based on our previous protocol [16]. To initiate the callogenesis process, the hypocotyl explants were carefully excised from *in vitro*-grown seedlings. A basal medium was prepared using MS [40] medium with 30 g/L sucrose and 7 g/L agar, and varying concentrations of phytohormones were subsequently added according to the specific treatment protocols; the medium’s pH was then adjusted to 5.7, followed by autoclaving at 121°C and 15 psi of pressure for 20 minutes. 35 mL of the prepared media were distributed in glass vessels. Then, 4 explants were cultured in each vessel.

A total of 33 treatments were applied, consisting of different concentrations of four phytohormones: Benzylaminopurine (BAP: 0, 0.5, 1, 1.5, and 2 mg/L), Kinetin (KIN: 0, 0.5, 1, 1.5, and 2 mg/L), α-Naphthaleneacetic acid (NAA: 0, 0.0.5, 0.1, 0.15, 0.2, 0.5, 1.5, and 2 mg/L), and Indole-3-butyric acid (IBA: 0, 0.0.5, 0.1, 0.15, 0.2, 0.5, 1.5, and 2 mg/L). The experimental design employed for this study was a completely randomized design with factorial arrangement. Each treatment was replicated seven times, resulting in a total of 231 experimental units. The cultures were maintained under controlled environmental conditions with a photoperiod of 55±4 μmolm^−2^s^−1^ and a temperature range of 25±3°C. After 5 weeks, the number of explants forming callus and the fresh weight of the callus tissue were recorded for each treatment. Callus formation rate was determined by calculating the percentage of explants that produced callus tissue. Callus fresh weight was measured by weighing the generated callus tissue using a precision balance. The obtained dataset was used to feed machine learning algorithms.

### Modeling procedure

**Fig 1:**
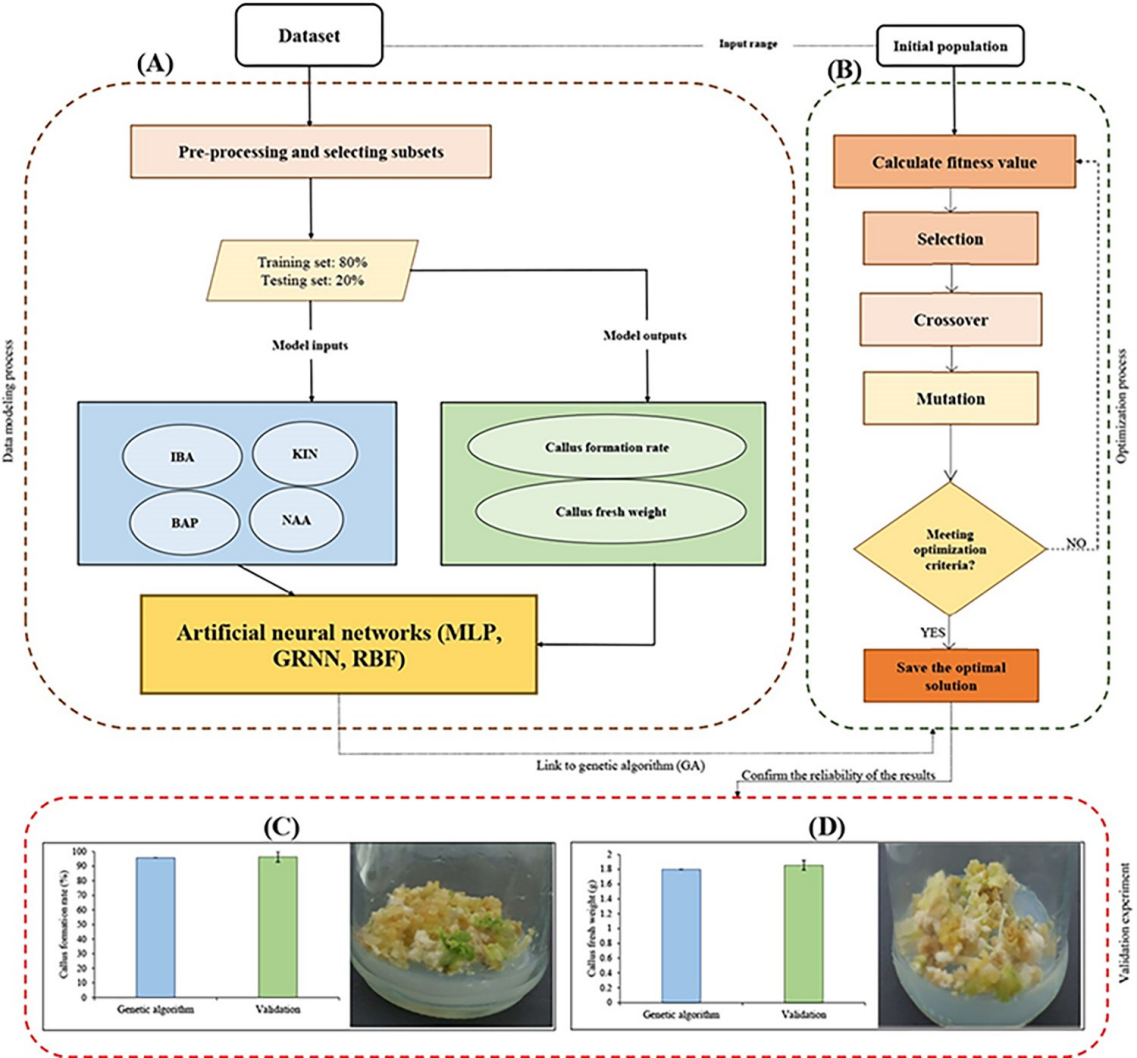
A schematic view of the step-by-step computational approach in the current study. (**A**) modeling and predicting callus formation rate and callus fresh weight (outputs) based on Benzylaminopurine (BAP), Indole-3-butyric acid (IBA), Kinetin (KIN), and α-Naphthaleneacetic acid (NAA) as inputs through three machine learning algorithms including generalized regression neural network (GRNN), multilayer perception (MLP) and radial basis function (RBF); (**B**) optimization process through a genetic algorithm (GA); and (**C-D**) confirming the results of the optimization process in the validation experiment for callus formation rate and callus fresh weight, respectively.

In this study, we employed three machine leaning algorithms (i.e., multilayer perceptron (MLP), radial basis function (RBF), and generalized regression neural network (GRNN)) for modeling and predicting callogenesis in petunia tissue culture. Our dataset consisted of 231 data points, where different concentrations of four phytohormones, namely BAP, KIN, NAA, and IBA, were considered as input variables (Fig 1A). The callus formation rate and fresh weight of callus were measured as the output variables. To construct the machine learning models, we randomly divided the dataset into a training set and a testing set using an 80:20 ratio (Fig 1A). Indeed, 80% of the data was used to train the ANN models, while the remaining 20% constituted the testing set for evaluating the model’s performance. Prior to training, the input variables were normalized to ensure equal weightage during the learning process. To ensure the robustness and generalizability of the models, we employed cross-validation techniques such as k-fold cross-validation, where the dataset was divided into k subsets, and the model was trained and validated multiple times using different combinations of training and testing sets. After training, the performance of the developed models was evaluated using the testing set. We assessed the accuracy of the predictions by calculating various metrics, including root mean squared error (RMSE), coefficient of determination (R^2^), and mean bias error (MBE).

### Multilayer perceptron (MLP)

The MLP is a feedforward artificial neural network (ANN) consisting of multiple layers, including an input layer, one or more hidden layers, and an output layer. It is known for its ability to capture complex non-linear relationships between input and output variables.

The architecture of the MLP network used in our study comprised an input layer, one hidden layer with 128 nodes, and an output layer. The input layer received the concentrations of four phytohormones, namely BAP, KIN, NAA, and IBA, as input variables. Each input variable was represented by a neuron in the input layer.

The hidden layer performed the intermediate processing of the input data. It consisted of multiple neurons, each connected to all neurons in the previous layer (input layer) and the subsequent layer (output layer). The hidden layer introduced non-linear transformations to the input data through the application of sigmoid activation function, which added non-linearity to the network.

The output layer produced the predicted output values, which in our study were the callus formation rate and fresh weight of callus. Similar to the hidden layer, the output layer also applied an activation function to transform the aggregated inputs from the hidden layer into the desired output format.

During the training phase, the MLP network adjusted its weights and biases using a process called backpropagation. Backpropagation involved propagating the error backward through the network, computing the gradients, and updating the weights and biases using optimization algorithms like stochastic gradient descent. This iterative process aimed to minimize the difference between the predicted output values and the actual output values, optimizing the network’s ability to model the callogenesis process accurately.

### Radial basis function (RBF)

The RBF network is a type of ANN known for its ability to approximate non-linear relationships and capture complex patterns in the data. The architecture of the RBF network consists of three main layers: the input layer, hidden layer, and output layer. Each layer performs specific functions to process the input variables and generate the desired output. The input layer of the RBF network receives the input variables, which in our study are the concentrations of BAP, KIN, NAA, and IBA. Each input variable is represented by a neuron in the input layer, where the values are fed forward to the hidden layer for further processing.

The hidden layer of the RBF network is responsible for transforming the input variables into a higher-dimensional feature space. Each neuron in the hidden layer computes the Euclidean distance between the input variables and its corresponding center, which represents a prototype or representative point in the feature space. The hidden layer neurons then apply a radial basis function, typically a Gaussian function, to calculate their activations based on the distance from the input variables to their respective centers.

The output layer of the RBF network generates the predicted output values, which in our study are the callus formation rate and fresh weight of callus. Each neuron in the output layer computes a weighted sum of the activations from the hidden layer, where the weights represent the influence of each hidden layer neuron on the final output. During the training phase, the RBF network adapts its parameters, including the centers and widths of the radial basis functions, to minimize the difference between the predicted output values and the actual output values. This was achieved through gradient descent, which adjusted the network’s weights to minimize the loss function.

### Generalized regression neural network (GRNN)

The GRNN is a specific type of RBF network that excels in interpolation tasks and is well-suited for pattern recognition and regression applications. The architecture of the GRNN consists of four main components: the input layer, pattern layer, summation layer, and output layer. Each component performs a specific function in processing the input data and producing the desired output. The input layer of the GRNN receives the input variables, which in our study are the concentrations of BAP, KIN, NAA, and IBA. The input layer acts as a conduit, passing the input values to the subsequent layers for further processing.

The pattern layer is responsible for storing the training patterns of the dataset. In our study, each training pattern comprises the input variables (phytohormone concentrations) and their corresponding output variables (callus formation rate and fresh weight of callus). The GRNN stores these patterns in its memory and uses them for subsequent computations.

The summation layer calculates the similarity between the input pattern and the stored training patterns. It employs the Gaussian kernel function to determine the weighted similarity between the input pattern and each stored pattern. The kernel function assigns higher weights to patterns that are closer to the input pattern, capturing the relationship between the input variables and the output variables. The output layer of the GRNN generates the predicted output values. It uses the weighted similarities calculated in the summation layer to estimate the output values based on the training patterns stored in the pattern layer. The output layer applies a weighted averaging mechanism, where the predicted output values are weighed by their respective similarities to the input pattern. During the training phase, the GRNN adjusts the weights of the network to minimize the difference between the predicted output values and the actual output values. This process is accomplished through a combination of supervised learning and memory-based reasoning, leveraging the stored training patterns to guide the learning process.

### Sensitivity analysis

In our study, we conducted a sensitivity analysis to assess the influence and relative importance of the input variables on the output variables in the context of callogenesis in petunia tissue culture. This analysis was performed through the calculation of the Variable Sensitivity Ratio (VSR), a widely used measure to quantify the sensitivity of a model’s output with respect to changes in the input variables. To determine the VSR values, we utilized the dataset consisting of different concentrations of four phytohormones (BAP, KIN, NAA, and IBA) as input variables, while the callus formation rate and fresh weight of callus were considered as the output variables. Initially, we trained the machine learning model, MLP in our case, using the entire dataset and obtained the corresponding predictions for the output variables. Subsequently, we performed a systematic variation of the input variables within a predetermined range while keeping the remaining inputs constant. For each variable, we incrementally modified its value and recorded the resulting changes in the output variables. This process allowed us to observe the sensitivity of the output variables to variations in the individual input variables. The VSR was calculated by dividing the absolute change in the output variable by the corresponding change in the input variable. By analyzing the VSR values for each input variable, we quantified the extent to which variations in each phytohormone concentration influenced the callus formation rate and fresh weight of callus. Higher VSR values indicated a stronger sensitivity, demonstrating that changes in the corresponding input variable had a more pronounced impact on the output variables.

### Genetic algorithm (GA)

In the current study, we linked GRNN as the most accurate model in our study to GA for the optimization process. Indeed, we employed GA to determine the optimal concentrations of BAP, KIN, NAA, and IBA for maximizing callogenesis parameters (i.e., callus formation rate and callus fresh weight) in petunia tissue culture. GA is a powerful optimization technique inspired by the process of natural selection and evolution. It is particularly well-suited for solving complex optimization problems with a single objective. The architecture of the GA used in our study consisted of several key components: the population, fitness function, selection mechanism, crossover operator, mutation operator, and termination criteria (Fig 1B). The population represented a set of potential solutions, with each solution corresponding to a specific combination of phytohormone concentrations. In our case, the concentrations of BAP, KIN, NAA, and IBA formed the variables of each solution within the population. The fitness function evaluated the performance or suitability of each solution in the population. In our study, the fitness function measured the callus formation rate resulting from each combination of phytohormone concentrations. The higher the callus formation rate, the higher the fitness score assigned to the corresponding solution. The selection mechanism determined which solutions would be selected as parents for reproduction in the next generation. Roulette wheel selection as one of the most powerful selection methods was used in the current study. Roulette wheel selection ensures that solutions with higher fitness scores have a higher probability of being selected as parents. The crossover operator facilitated the recombination of genetic material from selected parents to produce offspring. In our study, the crossover operation involved combining the concentrations of phytohormones from two parent solutions to create new solutions representing potential combinations of concentrations. The mutation operator introduced random changes or perturbations to the genetic material within the solutions. This randomness helped to explore the search space and prevent premature convergence to suboptimal solutions. In our case, the mutation operator modified the concentrations of phytohormones in the solutions to introduce novel combinations. The termination criteria determined when the GA should stop searching for optimal solutions. Common termination criteria include reaching a maximum number of generations, achieving a desired fitness threshold, or reaching a predefined computational time limit.

We utilized previously published Matlab codes [41] to develop ANN and GA algorithms, as well as to conduct sensitivity analysis in our study.

### Validation experiment

The reliability of the predicted-optimized results obtained through the genetic algorithm was evaluated through a validation experiment conducted in the laboratory. The validation experiment aimed to confirm the accuracy and reproducibility of the optimized combinations of phytohormones in achieving high callus formation rates and callus fresh weights. A total of seven replications were performed to ensure robustness and statistical significance. For each replication, the optimized phytohormone combinations determined by the genetic algorithm were used as treatment groups. Specifically, the combination of 1.31 mg/L BAP, 1.02 mg/L KIN, 1.44 mg/L NAA, and 1.70 mg/L IBA was tested for callus formation rate, while the combination of 1.39 mg/L BAP, 0.81 mg/L KIN, 1.22 mg/L NAA, and 1.60 mg/L IBA was evaluated for callus fresh weight (Fig 1C and 1D). The plant material and the tissue culture procedures, including explant preparation, media preparation, and incubation conditions, followed standard protocols as described for the first trial experiment. The data collected from the validation experiment were analyzed statistically to compare the results with the predicted-optimized outcomes of the genetic algorithm.

## Results

### Effects of phytohormones on petunia callogenesis

In this study, we investigated the effects of different concentrations of phytohormones, including BAP, KIN, NAA, and IBA, on callus formation rate and fresh weight of callus in petunia tissue culture. Our findings revealed significant variations in callus formation and growth in response to different hormone concentrations (Table 1). Notably, the absence of phytohormones in the culture medium resulted in the complete absence of callus formation. This highlights the essential role of exogenous hormones in promoting callus induction in petunia.

**Table 1:** Effects of different concentrations of various phytohormones on callus formation rate and callus fresh weight of petunia.

BAP (mg/L)	KIN (mg/L)	NAA (mg/L)	IBA (mg/L)	Callus formation rate (%)	Callus fresh weight (g)
0	0	0	0	0.00±0.000	0.00±0.000
0.5	0	0.05	0	46.43±8.502	1.46±0.054
0.5	0	0.5	0	75.00±7.715	1.47±0.026
0.5	0	0	0.05	53.57±12.711	1.24±0.116
0.5	0	0	0.5	64.29±5.051	1.21±0.076
1	0	0.1	0	75.00±7.715	1.43±0.036
1	0	1	0	85.71±5.051	1.50±0.039
1	0	0	0.1	78.57±3.571	1.52±0.079
1	0	0	1	89.29±5.051	1.67±0.026
1.5	0	0.15	0	67.86±4.611	1.24±0.094
1.5	0	1.5	0	89.29±5.051	1.65±0.061
1.5	0	0	0.15	85.71±5.051	1.60±0.046
1.5	0	0	1.5	92.86±4.611	1.79±0.035
2	0	0.2	0	71.43±12.711	1.31±0.220
2	0	2	0	67.86±13.041	1.12±0.212
2	0	0	0.2	71.43±12.711	1.12±0.202
2	0	0	2	89.29±5.051	1.69±0.054
0	0.5	0.05	0	53.57±10.102	1.25±0.212
0	0.5	0.5	0	82.14±4.611	1.48±0.052
0	0.5	0	0.05	64.29±12.023	1.27±0.228
0	0.5	0	0.5	75.00±7.715	1.39±0.032
0	1	0.1	0	75.00±5.455	1.46±0.059
0	1	1	0	82.14±4.611	1.59±0.077
0	1	0	0.1	67.86±7.143	1.51±0.069
0	1	0	1	57.14±10.514	1.17±0.199
0	1.5	0.15	0	78.57±3.571	1.38±0.084
0	1.5	1.5	0	53.57±10.102	1.10±0.192
0	1.5	0	0.15	75.00±5.455	1.60±0.070
0	1.5	0	1.5	82.14±4.611	1.58±0.014
0	2	0.2	0	57.14±10.514	1.20±0.208
0	2	2	0	85.71±7.435	1.53±0.008
0	2	0	0.2	42.86±11.845	1.08±0.278
0	2	0	2	71.43±12.711	1.34±0.225

The values in each column represent means ± Standard error.

BAP: Benzylaminopurine; IBA: Indole-3-butyric acid; KIN: Kinetin; NAA: α-Naphthaleneacetic acid.

Among the tested phytohormones, BAP and IBA demonstrated a pronounced effect on callus formation. The highest callus formation rate of 92.86±4.611% was achieved with a combination of 1.5 mg/L BAP and 1.5 mg/L IBA (Table 1). This concentration resulted in a substantial increase in callus initiation and development. Additionally, the fresh weight of the callus reached 1.79±0.035 g, indicating robust growth and biomass accumulation (Table 1).

In addition, varying concentrations of KIN and NAA exhibited different effects on callus formation rate or fresh weight of callus. 2 mg/L KIN along with 2 mg/L NAA resulted in higher callus formation rate (85.71±7.435%) in comparison with other concentrations of these phytohormones (Table 1). Overall, our results demonstrate that the presence and specific concentrations of phytohormones significantly influence callus formation in petunia tissue culture. The combination of 1.5 mg/L BAP and 1.5 mg/L IBA emerged as the most effective hormonal treatment, yielding the highest callus formation rate and fresh weight of callus. These findings provide valuable insights for the optimization of tissue culture protocols and callus induction strategies in petunia, contributing to the advancement of plant biotechnology applications in this economically important ornamental plant species.

### Evaluating and comparing different machine learning algorithms

In our study, we evaluated and compared the performance of three machine learning algorithms (i.e., MLP, GRNN, and RBF) through assessment criteria (i.e., R^2^, RMSE, and MBE) for predicting callus formation rate and callus fresh weight in petunia tissue culture (Table 2).

**Table 2:** Comparison statistics of different machine learning algorithms including MLP, GRNN, and RBF for modeling and predicting callogenesis rate and callus fresh weight of petunia.

Model	Measured parameter		Training			Testing	
		R^2^	RMSE	MBE	R^2^	RMSE	MBE
MLP	Callus formation rate	0.829	8.914	0.038	0.801	9.525	0.824
	Callus fresh weight	0.861	0.594	0.005	0.827	0.675	0.008
GRNN	Callus formation rate	0.859	6.939	0.026	0.837	7.178	0.049
	Callus fresh weight	0.889	0.315	0.002	0.846	0.363	0.004
RBF	Callus formation rate	0.831	8.563	0.033	0.811	9.131	0.806
	Callus fresh weight	0.866	0.562	0.005	0.851	0.641	0.008

GRNN: generalized regression neural network; MBE: mean bias error; MLP: multilayer perceptron; R^2^: coefficient of determination; RBF: radial basis function; RMSE: root mean squared error.

The MLP model exhibited a strong predictive capability for callus formation rate and callus fresh weight, with an R^2^ value of 0.801 and 0.827, respectively, in testing sets (Table 2). This indicates that approximately 80.1% and 82.7% of the variability in the callus formation rate and callus fresh weight, respectively, could be explained by the input variables. The low RMSE values of 9.525 and 0.675 further indicate the small average difference between the predicted and actual callus formation rate and callus fresh weight values, respectively (Table 2). Additionally, the MBE values of 0.824 and 0.008 indicate a slight positive bias in the predictions, indicating a slight tendency to overestimate the callus formation rate and callus fresh weight, respectively (Table 2). Overall, the MLP model demonstrated good performance in capturing the underlying patterns and predicting the callus formation rate and callus fresh weight in petunia tissue culture.

**Fig 2:**
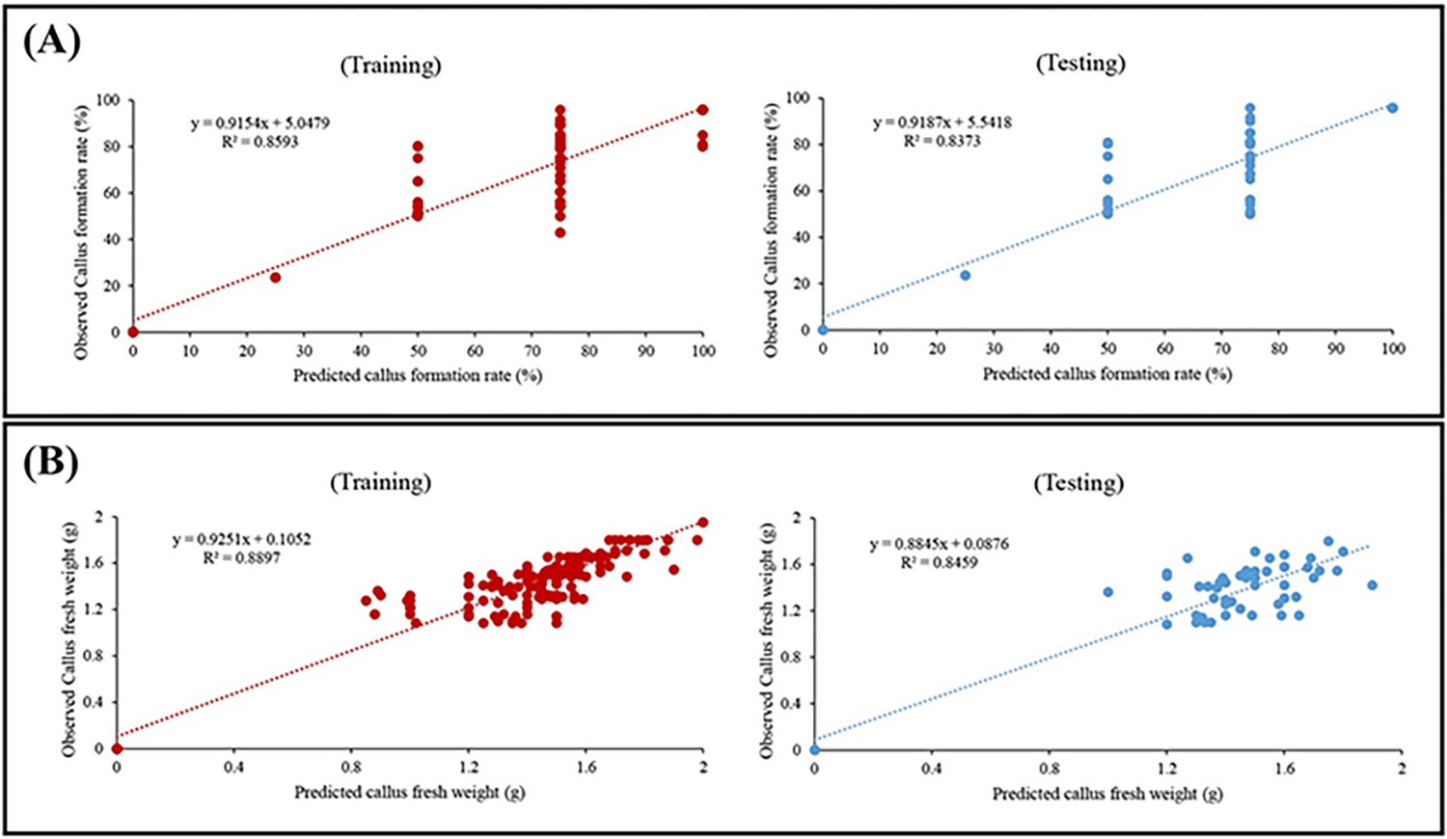
Scatter plot of observed vs. predicted values of (**A**) callus formation rate and (**B**) callus fresh weight obtained by generalized regression neural network (GRNN) in both training and testing sets.

The GRNN model also demonstrated favorable performance in predicting the callus formation rate and callus fresh weight, achieving R^2^ values of 0.837 and 0.846, respectively, on the testing sets (Table 2). This indicates that approximately 83.7% and 84.6% of the variability in the callus formation rate and callus fresh weight, respectively, can be attributed to the input variables (Fig 2). Compared to the MLP model, the GRNN model exhibited lower RMSE values of 7.178 and 0.363, indicating a smaller average difference between the predicted and actual values for callus formation rate and callus fresh weight, respectively (Table 2). Moreover, the MBE values of 0.049 and 0.004 for the callus formation rate and callus fresh weight, respectively (Table 2), indicate a slight positive bias, although it is smaller than that observed in the MLP model. These findings clearly indicate that the GRNN model outperformed the MLP model in terms of capturing the variability and accurately predicting the callus formation rate and callus fresh weight in petunia tissue culture.

Similarly, the RBF model demonstrated promising performance for callus formation rate and callus fresh weight, with an R^2^ values of 0.811 and 0.831, respectively (Table 2). This indicates that approximately 81.1% and 83.1% of the variability in the callus formation rate and callus fresh weight, respectively, could be explained by the input variables. The RMSE values of 9.131 and 0.641 indicate a comparable average difference between the predicted and actual callus formation rate and callus fresh weight values, respectively (Table 2), compared to the MLP model. The MBE values of 0.806 and 0.008 for the callus formation rate and callus fresh weight, respectively, suggest a slight positive bias, similar to the MLP model (Table 2). These results indicate that the RBF model also performed well in capturing the patterns and predicting the callus formation rate and callus fresh weight in petunia tissue culture.

Overall, the results of our evaluation indicate that all three machine learning algorithms, MLP, GRNN, and RBF, have demonstrated their effectiveness in modeling and predicting the callogenesis in petunia tissue culture. The GRNN model exhibited slightly better performance in terms of R^2^, RMSE, and MBE values, followed closely by the RBF and MLP models (Table 2). These findings provide valuable insights into the suitability and accuracy of these machine learning algorithms for predicting callus formation rate in petunia tissue culture applications.

### The importance of each phytohormone on petunia callogenesis

The sensitivity analysis conducted in our study revealed important insights into the impact of different phytohormones on callogenesis parameters, specifically the callus formation rate and callus fresh weight in petunia tissue culture. The analysis determined the relative importance of each phytohormone in influencing these parameters. The results indicated that among the four phytohormones studied, IBA exhibited the highest importance, followed by NAA, BAP, and KIN (Table 3). These findings provide valuable insights into the hierarchy of phytohormone importance in the regulation of callogenesis parameters in petunia tissue culture. The dominance of IBA highlights its critical role in promoting callus formation and growth, while the contributions of NAA, BAP, and KIN emphasize the involvement of auxins and cytokinins in the callogenesis process. These results contribute to our understanding of the underlying mechanisms governing tissue culture and provide a foundation for optimizing phytohormone concentrations to enhance callogenesis efficiency in petunia tissue culture protocols.

**Table 3:** Determination of the importance of each phytohormone on callus formation rate and callus fresh weight of petunia through sensitivity analysis.

Output	Item	BAP	KIN	NAA	IBA
Callus formation rate	VSR	1.151	1.082	1.1677	1.242
	Rank	3	4	2	1
Callus fresh weight	VSR	1.074	1.073	1.109	1.231
	Rank	3	4	2	1

BAP: Benzylaminopurine; IBA: Indole-3-butyric acid; KIN: Kinetin; NAA: α-Naphthaleneacetic acid; VSR: Variable Sensitivity Ratio.

### Optimization of the phytohormone concentrations for maximizing callogenesis

The present study aimed to optimize the callus formation rate and callus fresh weight in a tissue culture system using a genetic algorithm. The optimization process involved the manipulation of four phytohormones (i.e., BAP, KIN, NAA, and IBA). Through the genetic algorithm, the combination of 1.31 mg/L BAP, 1.02 mg/L KIN, 1.44 mg/L NAA, and 1.70 mg/L IBA was identified to yield a remarkable callus formation rate of 95.83% (Table 4). Additionally, another combination of 1.39 mg/L BAP, 0.81 mg/L KIN, 1.22 mg/L NAA, and 1.60 mg/L IBA resulted in a substantial callus fresh weight of 1.80 g (Table 4). These findings highlight the effectiveness of the genetic algorithm in optimizing the tissue culture system for enhanced callus formation and biomass accumulation. The results provide valuable insights for the development of efficient protocols for plant tissue culture and can contribute to the improvement of various applications such as plant propagation and genetic transformation.

**Table 4:** Determination of the optimal concentrations of phytohormones for maximizing the callus formation rate and callus fresh weight of petunia through genetic algorithm.

Fitness function	BAP	KIN	NAA	IBA	Predicted value
Callus formation rate	1.31	1.02	1.44	1.70	95.83%
Callus fresh weight	1.39	0.81	1.22	1.60	1.80 g

BAP: Benzylaminopurine; IBA: Indole-3-butyric acid; KIN: Kinetin; NAA: α-Naphthaleneacetic acid.

### Confirming the results of the optimization process through the validation experiment

The optimized combinations of phytohormones obtained through the genetic algorithm were further validated in a laboratory experiment to assess the reliability of the predicted results. The results of the validation experiment demonstrated that there was no significant difference between the validation results and the optimized results obtained through the genetic algorithm for both callus formation rate (96.43±3.571) (Fig 1C) and callus fresh weight (1.86±0.065) (Fig 1D). This confirms the reliability and accuracy of the genetic algorithm in predicting optimal PGR combinations for achieving high callus formation rates and callus fresh weights. The validation experiment provides strong support for the effectiveness of the genetic algorithm as a powerful tool in optimizing tissue culture systems. These findings have practical implications for the development of efficient protocols for plant tissue culture, enabling enhanced callus production and biomass accumulation for various applications such as plant propagation and genetic transformation.

## Discussion

Callus formation plays a critical role in plant regeneration, genetic transformation, and secondary metabolite production, making it a crucial step in plant biotechnology applications [31, 42, 43]. However, the factors influencing callus induction and growth in Petunia are multifaceted and interconnected, making it challenging to predict and control this process using traditional methods [20]. Therefore, the application of machine learning techniques offers a promising avenue for uncovering the underlying mechanisms and facilitating the optimization of callus formation protocols in Petunia. The reliability and accuracy of the machine learning approach in modeling and predicting various *in vitro* culture systems have been previously approved in different species such as cannabis [29, 32, 44–48], chickpea [49], ajowan [38], Prunus rootstock [50–52], chrysanthemum [27, 53–57], pear rootstock [58–60], *Passiflora caerulea* [28, 61], wheat [62], wallflower [63], walnut [64], and tomato [31].

In our study, we explored the performance of three different machine learning algorithms, namely MLP, RBF, and GRNN, for modeling and predicting callogenesis in petunia tissue culture. By considering different concentrations of four phytohormones (BAP, KIN, NAA, and IBA) as input variables and the callus formation rate and fresh weight of callus as output variables, we aimed to compare the effectiveness of these ML approaches in capturing the underlying patterns and optimizing the callogenesis process.

The results of our evaluation emphasize the efficacy of machine learning algorithms in capturing the relationships between phytohormone concentrations and callogenesis in petunia tissue culture. The GRNN model displayed the best overall performance, followed closely by the RBF and MLP models. These findings underscore the potential of machine learning techniques in optimizing tissue culture protocols by accurately predicting callogenesis. Furthermore, they provide valuable insights into the selection of appropriate algorithms for future studies and practical applications in petunia tissue culture. Firstly, the GRNN model is known for its ability to capture complex patterns and relationships in the data due to its non-linear nature [65]. It uses a radial basis function activation function, which allows it to approximate any continuous function with high accuracy [66]. This flexibility enables the GRNN model to effectively capture the intricate relationships between the concentrations of phytohormones and callogenesis [21] in petunia tissue culture. In contrast, the MLP model, while capable of modeling non-linear relationships, may struggle with highly complex patterns and may require more hidden layers and neurons to achieve similar performance [63]. Secondly, the GRNN model has a unique architecture that inherently possesses memory [62]. It can store and recall previously encountered patterns, which aids in generalization and prediction [21]. This memory-based approach allows the GRNN model to make predictions based on the similarities between the input patterns in the training set and the new data points [46]. This characteristic is particularly beneficial in the context of callogenesis prediction in petunia tissue culture, where historical data and patterns play a crucial role [20]. In contrast, the RBF model and MLP model lack this explicit memory capability, which may limit their ability to effectively capture long-term dependencies and generalize well to new data [66].

Furthermore, the GRNN model is relatively less prone to overfitting, a common issue in machine learning models [28]. Overfitting occurs when a model learns the noise and variability present in the training data rather than the underlying patterns and relationships [39]. The GRNN model’s smoothing parameter, also known as the spread, helps control the model’s generalization ability by determining the width of the radial basis functions [29]. This parameter acts as a regularization mechanism, preventing the model from fitting the training data too closely and thereby improving its ability to generalize to unseen data. This regularization property contributes to the robustness and accuracy of the GRNN model in predicting callogenesis [21].

By employing sensitivity analysis, we were able to identify the relative importance of the different phytohormones in regulating callogenesis [50, 67]. This information can guide the optimization of tissue culture protocols by focusing on the most influential variables. Additionally, the VSR values can assist in understanding the underlying mechanisms of callogenesis and provide valuable insights for further investigations and improvements in petunia tissue culture techniques. The sensitivity analysis was performed by calculating the sensitivity ratio for each phytohormone, which quantifies the contribution of each factor to the overall variation in the callogenesis parameters [29]. The analysis demonstrated that IBA had the most significant impact on both the callus formation rate and the callus fresh weight. This finding suggests that IBA plays a pivotal role in regulating the proliferation and growth of callus tissue in petunia. It may promote cell division and expansion, thereby enhancing the formation of callus tissue [9].

Following IBA, the sensitivity analysis revealed that NAA ranked second in terms of importance for callogenesis. NAA is known for its auxin-like properties, and its influence on callus formation rate and callus fresh weight underscores the significance of auxin in regulating tissue growth and differentiation [9]. BAP, a cytokinin, was found to be the third most important factor. Cytokinins are essential for cell division and differentiation, and their presence in the culture medium has been shown to stimulate callus initiation and growth [6–8, 68]. Finally, KIN, another cytokinin, exhibited the least importance among the four phytohormones studied. Although less influential than the other factors, KIN still contributed to the callogenesis process in petunia tissue culture.

The result of the optimization process through genetic algorithm showed that the combination of 1.31 mg/L BAP, 1.02 mg/L KIN, 1.44 mg/L NAA, and 1.70 mg/L IBA would result in a 95.83% callus formation rate. The results shed light on the role of these phytohormones in regulating callogenesis and provide valuable insights for optimizing tissue culture protocols in petunia propagation and plant improvement. Firstly, the effect of BAP on callus formation rate and fresh weight of callus was prominent in our study. Higher concentrations of BAP demonstrated a stimulatory effect on callogenesis, resulting in increased callus formation rate and fresh weight of callus [6–8]. This observation aligns with previous studies indicating the positive role of BAP as a cytokinin in promoting cell division and differentiation [6–8]. In addition to BAP, the influence of KIN on callus formation rate and fresh weight of callus was also examined. Our findings revealed that KIN exhibited a more nuanced effect compared to BAP. At lower concentrations, KIN had limited impact on callogenesis parameters. However, as the concentration increased, KIN demonstrated a dose-dependent response, leading to enhanced callus formation rate and fresh weight of callus. This suggests that KIN might play a role in modulating cell division and proliferation processes during callogenesis [9, 69, 70]. Moreover, our study explored the effect of NAA and IBA, two auxins, on callus formation rate and callus fresh weight in petunia. These findings align with previous studies [6–9] highlighting the role of auxins in promoting cell proliferation and callogenesis.

## Conclusion

Overall, our study provides valuable insights into the effect of different concentrations of BAP, KIN, NAA, and IBA on callus formation rate and fresh weight of callus in petunia tissue culture. The observed dose-dependent responses highlight the importance of carefully optimizing phytohormone concentrations to achieve desired callogenesis outcomes. These findings contribute to the advancement of tissue culture techniques for petunia propagation and plant improvement, facilitating the development of efficient protocols for large-scale production of superior plant materials. In addition, our study showcased the effectiveness of different machine learning algorithms, MLP, RBF, and GRNN, for modeling and predicting callogenesis in petunia tissue culture. In addition, the results of the current study revealed that GRNN outperformed MLP and RBF in terms of accuracy. Each algorithm demonstrated its unique strengths and limitations in capturing the intricate relationships between the input variables (phytohormone concentrations) and output variables (callus formation rate and fresh weight of callus). Further research and exploration of these ML techniques can contribute to advancing our understanding of the callogenesis process and facilitate optimization strategies in plant tissue culture applications.
